# Approach to Resource Management and Physical Strength Predict Differences in Helping: Evidence From Two Small-Scale Societies

**DOI:** 10.3389/fpsyg.2020.00373

**Published:** 2020-03-25

**Authors:** Marina Butovskaya, Michalina Marczak, Michał Misiak, Dmitry Karelin, Michał Białek, Piotr Sorokowski

**Affiliations:** ^1^Institute of Ethnology and Anthropology, RAS, Moscow, Russia; ^2^National Research University Higher School of Economics, Moscow, Russia; ^3^Institute of Psychology, University of Wrocław, Wrocław, Poland; ^4^Institute of Psychology, Norwegian University of Science and Technology, Trondheim, Norway; ^5^Institute of Geography, RAS, Moscow, Russia

**Keywords:** helping behavior, altruism, 2D:4D, hand grip strength, immediate return society, delayed return society, Yali, Hadza

## Abstract

Helping behavior is likely to have evolved to increase chances of survival of an individual and their group. Nevertheless, populations differ significantly in their eagerness to help, and little is known about populational and inter-individual determinants of these differences. Previous studies indicated that economic and physiological factors might influence helping behavior. The aim of the present study was to investigate the effects of approach to resource management of a society (immediate-return economy vs. delayed-return economy), prenatal androgenization (based on second-to-fourth digit ratio), and physical strength (based on hand grip strength) on helping behavior toward others. Helping was assessed in terms of both general eagerness to help and differential helping toward: (1) kin, (2) other group members indiscriminately, (3) friends, and (4) those from whom help was obtained in the past. Based on data collected in two small-scale societies (*n* = 306), we found that people in the egalitarian immediate-return society (the Hadza hunter-gatherers of Tanzania) displayed helping behavior significantly more often than people in a more stratified delayed-return economy (Yali horticulturalists of Papua). Additionally, our results revealed that physical strength was a significant predictor of helping behavior in women but not in men. We discuss our findings in the light of the adaptive value of helping behavior.

## Introduction

Helping is one of the fundamental aspects of altruistic behavior (Warneken and Tomasello, 2015). Spontaneous, unrewarded helping, even at a certain personal cost, is present in humans from an early age (Warneken, 2013). Current evidence indicates that non-human apes engage in basic forms of altruistic cooperation as well (Silk and House, 2016). Therefore, it seems plausible that helping behavior relies on innate processes that humans share with their closest evolutionary relatives (Warneken and Tomasello, 2006, 2015; Hamlin, 2013; Silk and House, 2016). At the same time, studies show great variability in the rates of helping behavior across populations (Levine et al., 2001, 2008; Knafo et al., 2009). Little is still known, however, about the roots of these inter-populational differences in helping behaviors. The current work aims to fill this gap.

A construct helpful in understanding why individuals help others without prospect of immediate return is inclusive fitness. This concept comes from evolutionary psychology, and looks at the number of offspring equivalents that a persons’ behavior helps to protect (Hamilton, 1964). This is an important distinction, since otherwise researchers only considered direct, personal fitness—the number of own offspring supported. Own child carries 1/2 of one’s genes, whereas sibling’s child carries 1/4 of one’s genes. Saving three sibling’s children is therefore beneficial over saving one own child. This helps to understand why people can engage in help—their relatives or tribesmen share part of their genes, and therefore further transmit individuals’ own genes.

Individuals may gain inclusive fitness directly or indirectly, through impact on their own reproduction (direct fitness effects), or influencing the reproduction of related individuals (indirect fitness effects). Cooperation could be favored between non-relatives, when individuals are preferentially aiding others who have helped them in the past, so-called “reciprocal altruism” (Trivers, 1971). Reciprocal altruism presents a mutually beneficial behavior, thus in reality, it is not a real altruistic behavior, but rather “reciprocity” (Alexander, 1974), or “reciprocal cooperation” (Axelrod and Hamilton, 1981). Accordingly, kin selection and reciprocal altruism may not be assumed as the leading explanations for cooperation or altruism (West et al., 2007). It is important to distinguish cooperation, which provides a direct fitness benefit to the individual that performs the behavior, outweighing the cost of such a behavior (Sachs et al., 2004), from the behavior, which provides indirect benefits, e.g., by helping close relatives to reproduce (Hamilton, 1964).

The place of altruism in human life is of special interest for anthropologists, given the growing amount of literature on cooperation. According to empirical approach, the behavior is altruistic, if it is costly to actor (Fehr and Fischbacher, 2003). Such behavior is analogous to the definition of helping or cooperation. Although, the same authors (Fehr and Fischbacher, 2004), propose the role of group social norms and third-party punishment in human societies. In contrast, the theoretical approach determines altruism as costly behavior, beneficial to all group members (Gintis, 2000; Boyd et al., 2003).

The existing cross-cultural evidence points to the crucial role of social environment in determining eagerness to help. In a large-scale experimental study that took place in 23 cities around the world, economic well-being was negatively correlated to help offered to strangers (Levine et al., 2001). Nevertheless, even though economic productivity was a significant predictor of helping rates as a general trend, the clusters of the most and least helpful populations were far from homogenous. What is more, a similar study in 24 cities across the United States yielded opposite results—this time residents of cities with greater economic productivity tended to be more helpful (Levine et al., 2008). This leaves the question of the relationship between economic factors and helping behavior open to further examination.

Helping might also be affected by a society’s approach to resource management. Societies are broadly classified into two major groups: (1) immediate-return societies, in which people obtain a direct and immediate return from their labor, such as a majority of hunter-gatherer societies, and (2) delayed-return societies, in which work is invested over extended periods of time before a yield is produced or consumed (Woodburn, 1982). Immediate-return societies do not have the capacity to accumulate resources, thus hierarchy in these societies is diffused (Woodburn, 1982). In consequence, immediate-return societies tend to be egalitarian (Lewis, 2017), whereas delayed-return economies are much more stratified. As the ability to accumulate resources increases, it becomes necessary to establish hierarchical structures of authority to distribute work and control resources (Woodburn, 1982).

These societies differ in the function of helping, with streamline effects on their behavior. More specifically, immediate-return societies are more generous, at least in terms of sharing food. A caveat to this is that sharing happens primarily by demand rather than by unsolicited giving (Ingold, 1980; Cashdan, 1985; Peterson, 1993). This has led to the development of the so-called “immediate-return morality” (1982). Such morality is characterized by an apparent indifference toward misfortunes of others and “a more pragmatic concern with being seen to do something when requested” (Peterson, 1993 p. 868). Such morality is driven by the fact that the individual hunter is not able to invest the yield of his labor in specific social relations. Given the high risk of uncertainty of food provisioning when there is no food storage, a person might make future claims for meat from other hunters. Such claims might not be limited to people who have previously received meat from them. The social norm in such societies should be thus to help anyone who asks for help.

Food sharing in Hadza begins at very young age. Food transfers increase approximately from the age 7–8, and occur with both related and unrelated children in the camp (Crittenden and Zes, 2015). Food sharing between Hadza children has been motivated by reciprocity, thus supporting recent claims that discrimination among kin might be linked with reciprocal altruism theory (Allen-Arave et al., 2008). The Hadza social networks show certain degree of skewed distribution, assortativity, transitivity, reciprocity, and homophily (Apicella et al., 2012). Reciprocity as a general basement of food sharing behavior (Jaeggi and Gurven, 2013) has been demonstrated in adult Hadza as well. Hadza respect and enjoy living in camps with affiliative people who work hard, and with whom they share a long history of mutual understanding. Food sharing and consumption data show that men channeled the foods they produced to their wives, children, and their consanguineal and affinal kin living in other households (Wood and Marlowe, 2013).

Helping in delayed-return societies is likely to serve a different function. In a society that is able to accumulate resources, reciprocity appears to play a vital role in altruistic behaviors (Gurven, 2004). In this context, sharing might be influenced by the drive to establish and maintain social status (Gurven, 2004). According to the competitive altruism hypothesis (Hardy and Van Vugt, 2006), individuals attempt to outcompete others in terms of pro-social behavior. Altruism enhances status and reputation and therefore yields benefits that would be unattainable otherwise. By contributing to a social group, an individual builds a reputation for generosity, making them more attractive as a future social partner (Hardy and Van Vugt, 2006; Anderson and Kilduff, 2009). The two types of societies can initiate helping for different reasons.

Finally, prenatal androgenization and physical strength can contribute to willingness to help. A growing body of evidence supporting this hypothesis comes from psychophysiological studies. Androgen receptor gene polymorphisms and testosterone level may enhance behaviors involved in obtaining and maintaining high social status and reproductive success in men (Butovskaya M. et al., 2015; Butovskaya M. L. et al., 2015). These factors may also enhance pro-social behaviors, i.e., generosity by both male and female individuals, as well as cooperation among male individuals (Boksem et al., 2013; Reimers and Diekhof, 2015; Dreher et al., 2016). Likewise, prenatal exposure to high levels of testosterone, as measured by the second to fourth digit ratio, 2D:4D, may be linked to helping behavior (Putz et al., 2004; Manning and Fink, 2008). Across studies, a more masculine digit ratio correlated positively to generosity (Millet and Dewitte, 2006, 2009; Van Honk et al., 2012; Brañas-Garza et al., 2013). This is in line with the competitive altruism hypothesis, as 2D:4D is considered a predictor of social status (Sluming and Manning, 2000; Manning and Taylor, 2001). Prenatal testosterone exposure affects not only 2D:4D, but also hand grip strength (Fink et al., 2006). Since it was found that upper body strength is a good predictor of hunting skills (Misiak et al., 2019) and hunting reputation in Hadza men (Apicella, 2014), we can expect a positive association between altruistic behaviors and hand grip strength. However, the link between masculinity and generosity might not be universal (Galizzi and Nieboer, 2015).

In this project, we explored the effects of the approach to resource management of a society (immediate-return economy vs. delayed-return economy), prenatal androgenization (based on 2D:4D ratio data), and physical strength (based on hand grip strength) on helping behavior. We further explored differences in helping toward different social partners in need: (1) kin, (2) other group members indiscriminately, (3) friends, and (4) those from whom help was obtained in the past. The way of life in traditional pre-industrial societies resembles the one in which humans evolved for most of their history. Therefore, the study of determinants of helping behavior in such populations can provide a better understanding of this phenomenon (Henrich et al., 2010). This is especially important because helping developed as an essential adaptation in human evolutionary history, possibly playing a major role in cooperative breeding (Crittenden and Marlowe, 2008).

We chose two different traditional societies for our study. The immediate-return Hadza hunter-gatherers of Tanzania (Woodburn, 1982; Marlowe, 2010; Apicella et al., 2012; Butovskaya M. L. et al., 2015) and the more delayed-return Yali horticulturalists of the Papuan highlands (Koch, 1974; Marczak et al., 2018; Sorokowski et al., 2020). We hypothesized that (H1) representatives of an immediate-return society, such as the Hadza, contrasted with a delayed-return society, such as the Yali, will be more eager to provide help toward others, no matter who the recipients of help are. Given the rising importance of reciprocity in delayed-return economies, we also hypothesize (H2) that the Yali will offer help primarily to those from whom help was obtained in the past. Moreover, we hypothesize that (H3) physically stronger individuals (i.e., those with higher HGS) will be more inclined to help others, and (H4) more masculine (i.e., lower 2D:4D) scores will be positively associated with helping behavior, in both men and women.

## Materials and Methods

### Participants

Data on the nomadic Hadza were collected in 2015–2018 in the Mang’ola region of Tanzania. The participants were 223 individuals (117 men) with mean age of approximately 35 years (range: 18–75 years). No data on the Hadza people settled in sedentary villages were included. Data from the Yali were collected in 2016 in the Yalimo highlands of Papua, a semi-independent province of Indonesia. The total sample included 83 individuals (49 men) with a mean age of approximately 40 years (range: 18–75 years).

The Hadza are a nomadic society of foragers living in northern Tanzania. They number approximately 1000–1500 individuals and live in mobile camps, each comprising an average of 30 people. Sexual division of labor is obviously expressed, with men and women being the hunters and gatherers, respectively (Marlowe, 2010; Jones, 2016). The Hadza are an example of an immediate-return egalitarian society (Butovskaya, 2013; Butovskaya M. L. et al., 2015). They do not produce any food items and they do not accumulate food to be consumed in the delayed future. Big-game meat, the main item widely distributed among camp members, cannot be stored by hunters for long periods because of the hot climate and absence of conservation technology (Marlowe, 2010; Apicella et al., 2012).

The Yali pursue traditional horticulturalist lifestyle in the harsh mountainous environment in the Eastern Highlands of the Baliem Valley, West Papua, Indonesia (Koch, 1974). They produce most of their food in gardens, where they practice shifting cultivation (Koch, 1974). Their society is strongly male-dominated and polygyny is prevalent (Sorokowski et al., 2013). Food supply labor is divided between the sexes, and men tend to occasionally hunt small marsupials and mammals, while women harvest and cultivate. Although there is no official leadership in the Yali, they tend to distinguish some physically fit and charismatic males, “big-men,” who are able to signal their wealth by providing pigs for feasting (Koch, 1974; Sorokowski et al., 2013).

### Procedure

All participants provided informed consent before study inclusion, and they were instructed they could quit the procedure at any time. Most of the informants were illiterate, thus the consent was oral. The study complied with the Declaration of Helsinki on Biomedical Research Involving Human Subjects. Data were collected in the form of interviews in Ki-Swahili (Hadza) and the Yali language (Yali). The local assistants read all questions aloud in one-to-one dialogs, and further explanations were provided if necessary. More details are provided below. No other group members were allowed to stay nearby, in this way we tried to avoid at least some biases associated with social desirability.

#### Digit Ratio Assessment

The second and fourth digits of participants were measured with a digital Vernier caliper with.01 mm accuracy—we took measures of the second and fourth hand digit from a mid-point on the ventral crease proximal to the palm to the tip of the finger (Manning et al., 1998). Participants who reported injuries or deformities of at least one of these digits were excluded from the statistical analysis. Each measurement was collected twice from each participant. The means of the first and the second measurement of right and left 2D and 4D, as well as the right and left 2D:4D ratios were calculated following the procedure described by Manning et al. (1998) and Manning (2008). Intra-class correlation coefficients (ICCs) were calculated to assess the reliability of right and left hand 2D:4D between the two measurements. The ICCs were 0.93 and 0.96 for the right 2D:4D and left 2D:4D for the Hadza sample, and 0.86 and 0.97 for the Yali sample, respectively.

#### Hand Grip Strength Assessment

We measured maximum voluntary contraction (MVC) of the grip flexors, commonly known as hand grip strength using the Harpenden spring dynamometer (Balogun et al., 1991). Each participant was instructed to take a comfortable position and squeeze the dynamometer with their dominant hand as hard as they could, with a dominant hand lifted. We took measures three times. ICCs between measurements were high for both hands (0.98 and.99 for the Hadza and 0.82 and 0.87 for the Yali, for right and left hand, respectively). To analyze hand grip strength, we used the average of all three grips.

#### Helping Behavior Assessment

To assess helping behavior among participants, we asked them to estimate their involvement in helping others using four statements: (1) “I struggle to help my relatives if they are in need” (kin altruism); (2) “I can’t refuse if people ask for help” (helping in-group members); (3) “I help my friends if they have problems” (helping friends); (4) “I help those who helped me in the past” (direct reciprocity). Possible answers were rated on a Likert scale, from 1 (“never”) to 5 (“always, or regularly”). Initially the statements looked as follows: (1) “I help my relatives”; (2) “I help other people (in-group members)”; (3) “I help my friends”; (4) “I help those who helped me in the past.” Each of these statements addressed a certain type of altruistic behavior close to the specifications presented by Nowak (2006). We decided to present the questions in current format, because in a small pilot study obtained a lot of comments from our respondents, asking to specify in which case and if a potential benefiter has been asking for help or not. Before the main study, we tested the level of understanding of these statements among a group of Hadza and Yali people to exclude misunderstandings. In most cases, these questions were accompanied by further explanations and examples suitable to each society, e.g., helping with a child (both societies: caring for a child when mother is busy; bringing food or water for a mother with a new born baby); helping with carring meat after hunting (men in both societies); helping with cooking (both societies); helping with house construction and cleaning a new field (Yali); protecting in disputes (both men and women, in both societies); protecting against strangers (Yali men). Sharing on demand (under pressure) is something basic for Hadza society. Direct address with a request for help also seems common.

To verify whether participants comprehended the questions, we asked them to provide examples of particular activities and memories of situations when they helped. A short list of questions about helping behavior was used to obtain the most precise answers possible while maintaining respondent attention and interest in the interview. The Cronbach’s alpha values for total helping were reliable (in the Hadza: Cronbach’s α = 0.71, *n* = 223; in the Yali: Cronbach’s α = 0.79, *n* = 83).

Apart from society type, we also recorded information about participants’ sex and age. We decided to divide the sample into age groups, as many respondents did not know their exact age; thus, they were approximated in accordance with the informants’ personal information about their life events and their groupmates’ comments. Consequently, we divided the participants into six age groups, from 18–19 years (group 1) to 60 years and older (group 6).

## Results

Statistical analyses were performed using R program, version 3.4.3 (R Core Team, 2017). We used multilevel modeling to estimate the general likelihood of helping as predicted by the type of society, physical strength (mean HGS), digit ratio (mean 2D:4D), sex, and age group. We also inserted first-order interactions of type of society, physical strength, digit ratio with sex. We controlled for the type of helping behavior.

**FIGURE 1:**
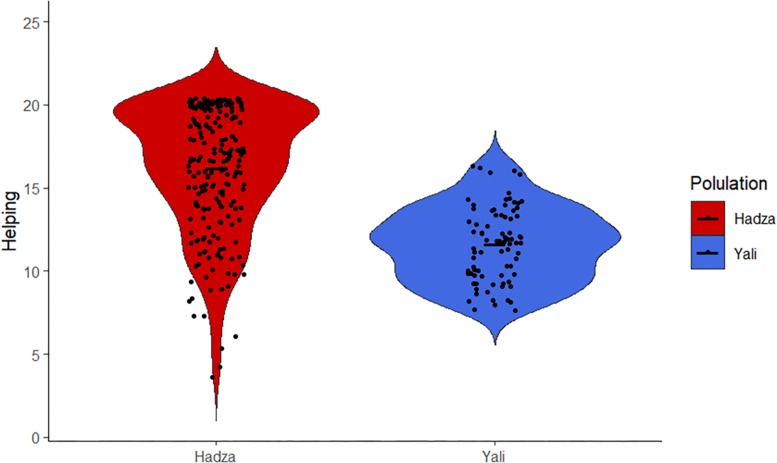
Differences in helping between Hadza and Yali. Note: Data represent summary score of all items measuring helping. Points are jittered to avoid overplotting.

**FIGURE 2:**
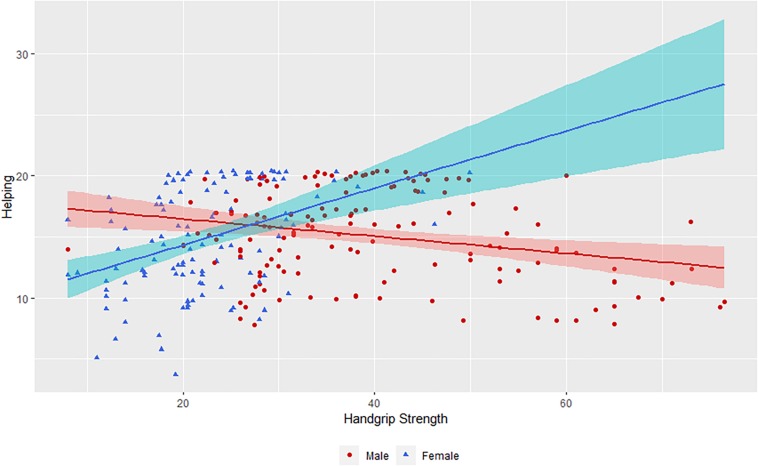
Relationship between handgrip strength, helping split by participants’ sex. Note: Data represent summary score of all items measuring helping. Shaded area corresponds to 95% confidence intervals. Points are jittered to avoid overplotting.

From the model, the only predictors of helping were ethnicity and handgrip by sex interaction (Table 1). More specifically, Hadza were far more willing to help than Yali (Figure 1). For the interaction, greater handgrip was associated with increased helping only in women, but not in men (Figure 2). The model explained the self-reported helping behavior well, conditional *R*^2^ = 0.510.

**TABLE 1:** Predictors of helping among Hadza and Yali.

	Help				
Predictors	Estimates	CI	Statistic	df	p
(Intercept)	5.518	2.209−8.827	3.269	252.967	**0.001**
age_group: age_group2	–0.318	−0.772−0.136	–1.373	252.972	0.170
age_group: age_group3	–0.044	−0.501−0.412	–0.191	252.972	0.849
age_group: age_group4	–0.129	−0.600−0.341	–0.539	252.972	0.590
age_group: age_group5	–0.242	−0.743−0.259	–0.945	252.972	0.344
age_group: age_group6	–0.438	−1.021−0.145	–1.473	252.972	0.141
ethnic: ethnic2	–1.286	−1.601to−0.972	–8.011	252.972	**<0.001**
ethnic2:sex2	0.321	−0.156−0.798	1.319	252.972	0.187
hand_gripmean	–0.000	−0.011−0.010	–0.058	252.972	0.954
hand_gripmean:sex2	0.039	0.016−0.062	3.321	252.972	**0.001**
mean2d_4d	–1.152	−4.517−2.212	–0.671	252.972	0.502
mean2d_4d:sex2	–0.606	−6.046−4.834	–0.218	252.972	0.827
sex: sex2	–0.469	−5.850−4.912	–0.171	252.972	0.864
**Random effects**					
σ^2^	0.75				
τ_00 id_	0.40				
τ_00 type_help_	0.00				
ICC	0.35				
N_id_	253				
N_type_help_	4				
Observations	1012				
Marginal *R*^2^/conditional *R*^2^	0.250/0.510				

*Bold value means statistically significant at *p* < 0.05.*

Further exploratory analyses, separating different types of helping behavior yielded no effects of helping type, nor its interactions (see Supplementary Material for exact coefficients). For this analysis, we had to exclude age from predictors, because the model was rank deficient, and age was not theoretically interesting. Moreover, the sex by handgrip interaction was now not significant. Therefore, our findings sold regardless of who the recipient of the help is.

## Discussion

The current study sought to examine the effects of the approach to resource management, prenatal androgenization, and physical strength on helping behavior toward others. We hypothesized that people in an immediate-return society would be more eager to provide help toward others, no matter who the recipients of help were. Moreover, we predicted that representatives of a delayed-return society would be more engaged in reciprocal helping than in other types of helping. Additionally, our hypotheses were that physical strength and an androgenized hormonal profile will be linked to higher rates of helping behavior.

Our data collected from two small-scale societies differing in their approach to resource management revealed significant differences in personal inclination for helping. Thus, our first hypothesis was confirmed: for all four types of helping, the immediate-return Hadza respondents rated themselves significantly higher than the delayed-return Yali participants, and this effect was independent of gender. It seems likely that, to cope with the extreme mutual interdependence of group-members in the immediate-return societies, social norms emphasizing cooperation, generosity, and sharing are developed (Christen et al., 2014). Sharing practices in the Hadza also illustrate this statement. For example, meat sharing in this society is practically indiscriminate for ecological reasons such as the unpredictability of game acquisition and cultural norms associated with meat consumption (Hawkes et al., 1989; Widlok and Tadesse, 2005; Marlowe, 2010). Big game, as demonstrated by Hawkes et al., is a kind of public good, and “instead of a set of exchanges with the hunter, the process of distribution is more like appropriation from the public domain” (Hawkes et al., 1989, p. 131). Our study shown that not only sharing practices, but also helping behavior is common and indiscriminate in this immediate-return society.

The second hypothesis was not confirmed: both tribes helped indiscriminately. Indiscriminate helping may be a basic necessity for group survival both in immediate-return societies and in delayed-return societies. Whereas nomadic hunter-gatherer way of life requires helping more people more often, in a society that has the ability to accumulate resources, helping rates drop, and altruistic behavior becomes a way of “costly signaling” of one’s qualities and status (Gintis et al., 2001).

First of all, it must be noted that in both sexes approach to resource management of a society was by far the firmest predictor of helping behavior (no sex by ethnicity interaction was observed). Nevertheless, we found that physical strength (as measured by handgrip strength) predicted reciprocal helping behavior in women, but not in men. In women, such association was positive meaning that individuals with higher physical strength reciprocated more. Similarly, female but not male handgrip strength was associated with greater reproductive success in indigenous Namibians (Atkinson et al., 2012). To the best of our knowledge, this is the first study that has presented a link between physical strength and helping behaviors. An indirect insight about this effect can be obtained through findings that handgrip strength correlates with inequality acceptance in men, but not in women (Petersen and Laustsen, 2019). Hence, stronger man support more competitive worldview, an association not found in females. Our findings suggest quite the opposite happens in women, where greater strength implies greater helping in traditional societies. We suggest that the association between hand grip strength and helping behavior may be subject to positive selection in females, as helping is less costly and more beneficial for physically healthier and stronger individuals. At the same time, cultural norms among men may have influenced the lack of an association observed among women. For example, Hadza men’s religious ceremonies force them to share indiscriminately with all co-resident initiated men in the group (Marlowe, 2010).

Contrary to what we expected, helping in general was not associated with digit ratio in our samples. It was suggested that people with low 2D:4D are more likely to act cooperatively and less likely to act egoistically (Millet and Dewitte, 2006). However, other similar studies have shown mixed results (Kastlunger et al., 2010; Brañas-Garza et al., 2013). Therefore, the relationship between prenatal androgenization and helping is probably either weaker than expected in general or influenced by variations in cultural norms and social attitudes in the studied populations.

Our study has several limitations. Particularly, we did not observe real cases of helping behavior; instead, we based our conclusions on subjects’ answers about their helping behavior. We cannot exclude the possibility that our findings reflect cultural differences in social desirability. The way the subjects understand and answer questions may be culturally dependent. Some categories of individuals (e.g., young versus old) may want to appear more cooperative to the experimenters. However, given the gender- and age-independent differences obtained between groups, we suggest that our subjects provided more or less reliable information. Another limitation is that our study examined the determinants of helping behavior in small-scale societies based on only two samples. In order to make general claims about the influence of approach to resource management, prenatal androgenization, and physical strength on helping behavior, more research in traditional societies is needed.

Helping can be seen through the lens of social networks. For example, westerners’ social networks after they turn 30 are usually composed of their kin (Wrzus et al., 2013). We did not collect any data about the size and composition of the social network of the participants, neither in general, nor in relation to age. Still, some insight can be gained from our informal observations. Group membership in Hadza is in constant rotation, and it is more appropriate to view Hadza as being a part of the local population, rather than of a particular group. Although, certain preferences may be mentioned, as Hadza prefer to stay with relatives, particularly, relatives from wife’s side (Marlowe, 2010). Besides, Hadza are used to spent time in same-sex groups during daily activities. Such groups include individuals of all age cohorts. Hadza networks are characterized with assortativity, transitivity, reciprocity and homophily, and decay with geographic distance (Apicella et al., 2012). Thus in their basic characteristics, Hadza networks are similar to modern societies. Although, personal networks are larger in more individualistic countries compared to collectivistic (Hofstede, 1980; Triandis et al., 1988).

## Conclusion

Despite the limitations mentioned above, our study provides data confirming the idea that in small-scale societies, factors such as approach to resource management and physical strength may influence individual helping behavior. We demonstrated that such factors might be gender-specific. Particularly, we showed that strength was positively associated with decisions about helping others in women, but not in men. Future studies are needed to understand why physical strength was a predictor of helping others in women but not in men, and whether the same factors are associated with helping behavior in other societies. Given that strength is a proxy of health, it may also be reasonable to test whether stronger women in modern societies are more inclined to provide help. Most importantly, our study shown that differences in resource management in a human society seem to shape not only its social structure and political organization, but they also influence personal motivations for helping others.

## Data Availability Statement

The raw data and the R script are publicly available at https://osf.io/Y54JB/.

## Ethics Statement

The studies involving human participants were reviewed and approved by the Institutional Review Board of the Institute of Psychology, University of Wrocław. The patients/participants provided their informed consent to participate in this study.

## Author Contributions

MaB, MMi, and PS designed the study. MaB, DK, MMi, and PS collected the data. MiB and MMa analyzed the data. MMa, MaB, MMi, DK, MiB, and PS wrote the manuscript.

## Conflict of Interest

The authors declare that the research was conducted in the absence of any commercial or financial relationships that could be construed as a potential conflict of interest.
