# Supplementary material for: Approach to Resource Management and Physical Strength Predict Differences in Helping: Evidence From Two Small-Scale Societies
Source: Front Psychol. 2020 Mar 25;11:373. doi: 10.3389/fpsyg.2020.00373 (PMC7110700; doi:10.3389/fpsyg.2020.00373)
Supplement: Supplementary file 1 [file Table_1.DOCX]

**Supplementary analysis**

Predictors of helping behavior with additional control of the type of help recipient (type_help).

Predictors are coded:

Ethnic: 1 – Hadza, 2 – Yali

Sex: 1 – Male, 2 – Female

Type help: (1) kin, (2) other group members indiscriminately, (3) friends, and (4) those from whom help was obtained in the past

|  | **help** | | | | |
| --- | --- | --- | --- | --- | --- |
| *Predictors* | *Estimates* | *CI* | *Statistic* | *df* | *p* |
| (Intercept) | 5.825 | -0.335 – 11.985 | 1.853 | 253.301 | 0.064 |
| ethnic2:sex2:type_helpempathy2 | 0.002 | -0.655 – 0.660 | 0.006 | 759.000 | 0.995 |
| hand_gripmean | -0.009 | -0.168 – 0.150 | -0.113 | 253.001 | 0.910 |
| type_help: empathy2 | -0.200 | -0.445 – 0.045 | -1.602 | 759.000 | 0.109 |
| type_help: empathy3 | -0.053 | -0.297 – 0.192 | -0.422 | 759.000 | 0.673 |
| type_help: empathy4 | -0.053 | -0.297 – 0.192 | -0.422 | 759.000 | 0.673 |
| mean2d_4d | -1.979 | -8.333 – 4.376 | -0.610 | 253.001 | 0.542 |
| mean2d_4d:hand_gripmean | 0.020 | -0.145 – 0.185 | 0.238 | 253.001 | 0.812 |
| sex: sex2 | -0.006 | -0.353 – 0.341 | -0.032 | 622.787 | 0.974 |
| ethnic2:sex2 | 0.267 | -0.353 – 0.887 | 0.845 | 642.472 | 0.398 |
| ethnic: ethnic2 | -1.491 | -1.894 – -1.088 | -7.254 | 645.816 | **<0.001** |
| ethnic2:type_helpempathy4 | 0.292 | -0.137 – 0.720 | 1.335 | 759.000 | 0.182 |
| ethnic2:sex2:type_helpempathy3 | 0.199 | -0.459 – 0.857 | 0.593 | 759.000 | 0.553 |
| sex2:type_helpempathy2 | 0.150 | -0.212 – 0.512 | 0.812 | 759.000 | 0.417 |
| sex2:type_helpempathy3 | 0.040 | -0.322 – 0.402 | 0.217 | 759.000 | 0.828 |
| sex2:type_helpempathy4 | -0.110 | -0.472 – 0.252 | -0.595 | 759.000 | 0.552 |
| ethnic2:sex2:type_helpempathy4 | 0.152 | -0.506 – 0.810 | 0.453 | 759.000 | 0.651 |
| ethnic2:type_helpempathy2 | 0.048 | -0.381 – 0.476 | 0.219 | 759.000 | 0.827 |
| ethnic2:type_helpempathy3 | -0.186 | -0.615 – 0.242 | -0.853 | 759.000 | 0.393 |
| **Random Effects** | | | | | |
| σ^2^ | 0.74 | | | | |
| τ_00_ _id_ | 0.45 | | | | |
| ICC | 0.38 | | | | |
| N _id_ | 253 | | | | |
| Observations | 1012 | | | | |
| Marginal R^2^ / Conditional R^2^ | 0.225 / 0.520 | | | | |
